# Biochar amendment changes jasmonic acid levels in two rice varieties and alters their resistance to herbivory

**DOI:** 10.1371/journal.pone.0191296

**Published:** 2018-01-26

**Authors:** Muhammad Waqas, Raheem Shahzad, Muhammad Hamayun, Sajjad Asaf, Abdul Latif Khan, Sang-Mo Kang, Sopheap Yun, Kyung-Min Kim, In-Jung Lee

**Affiliations:** 1 School of Applied Biosciences, Kyungpook National University, Daegu, Republic of Korea; 2 Department of Agriculture Extension, Government of Khyber Pakhtunkhwa, Buner, Pakistan; 3 Department of Botany, Abdul Wali Khan University Mardan, Pakistan; 4 UoN Chair of Oman's Medicinal Plants & Marine Natural Products, University of Nizwa, Nizwa, Oman; RMIT University, AUSTRALIA

## Abstract

Biochar addition to soil not only sequesters carbon for the long-term but enhances agricultural productivity. Several well-known benefits arise from biochar amendment, including constant provision of nutrients, increased soil moisture retention, decreased soil bulk density, and sometimes the induction of systemic resistance against foliar and soil borne plant pathogens. However, no research has investigated the potential of biochar to increase resistance against herbivory. The white-backed plant hopper (WBPH) (*Sogatella furcifera* Horváth) is a serious agricultural pest that targets rice (*Oryza sativa* L.), a staple crop that feeds half of the world’s human population. Therefore, we investigated the (1) optimization of biochar amendment levels for two rice varieties (‘Cheongcheong’ and ‘Nagdong’) and (2) subsequent effects of different biochar amendments on resistance and susceptibility of these two varieties to WBPH infestation. Initial screening results for the optimization level revealed that the application of biochar 10% (w/w) to the rooting media significantly improved plant physiological characteristics of both rice varieties. However, levels of biochar amendment, mainly 1, 2, 3, and 20%, resulted in negative effects on plant growth characteristics. Cheongcheong and Nagdong rice plants grown with the optimum biochar level showed contrasting reactions to WBPH infestation. Specifically, biochar application significantly increased plant growth characteristics of Nagdong when exposed to WBPH infestation and significantly decreased these characteristics in Cheongcheong. The amount of WBPH-induced damage to plants was significantly lower and higher in Nagdong and Cheongcheong, respectively, compared to that in the controls. Higher levels of jasmonic acid caused by the biochar priming effect could have accumulated in response to WBPH infestation, resulting in a maladaptive response to stress, negatively affecting growth and resistance to WBPH in Cheongcheong. This study highlights the importance of investigating the effects of biochar on different rice varieties before application on a commercial scale to avoid potential crop losses.

## Introduction

Rice is at the top of globally important agronomic crops (*Oryza sativa* L.) and is a major dietary staple for half of the human population. The demand for rice is on the rise, particularly due to the steadily increasing populations of countries in Asia, Africa, and Latin America [1]. In China, rice production needs to be increased by approximately 20% by 2030 to meet local demand if rice consumption per capita remains at its present level [2]. Therefore, rice is grown over a huge area of cultivated fields worldwide. In India 43.86 million ha cropping area with diverse environmental conditions are utilized for rice cultivation [3]. However, rice crop production is affected by numerous biotic and abiotic factors such as insects, diseases, and other environmental stresses. Insect infestations in rice have recently become intensified across Asia, resulting in heavy yield losses [4]. Among these insects, the white-backed plant hopper (WBPH) (*Sogatella furcifera* Horváth, Homoptera: Delphacidae) appeared as a serious piercing-sucking pest responsible for damaging rice grown in Asian countries. The WBPH can rapidly devastate rice crops due to its long-distance migration ability, and it has caused sporadic famines in eastern Asia since ancient times. It became particularly conspicuous after the Green Revolution in the Southeast Asia region. Nymphs and adults damage the rice plants by feeding on the phloem. Additionally, they turn the crop into a meaningful source of transmitting major viral diseases, such as southern rice black-streaked dwarf virus. Important crop factors such as plant vigor, height, number of productive tillers, filled grains, and yield are subsequently negatively affected [5]. Moreover, if heavy WBPH infestation occurs at the tillering stage, complete necrosis or death of rice plants occurs—a condition is called “hopper burn” [6]. In rice agronomic practices, the management of WBPH infestation largely relies on chemical pesticides. However, this method has economic and environmental consequences, such as killing WBPH predators, and pesticide resistance in insects cannot be avoided. All of these factors lead to pest resurgence, including indiscriminate insecticide usage during early crop stages of recorded outbreaks of sap-sucking insects [7]. On the other hand, massive pesticide application adversely affects pests that are known to be beneficial to crop plants, as well as pose serious risks to human health and the environment. Rice plants have previously been transformed with *Bacillus thuringiensis* (Bt) cry genes for defense against WBPH [8]. Various genes delivering resistance against sap-sucking insects have been sorted out in the rice germplasm itself; however, the development of resistant varieties is a slow process [9].

The use of Bt rice is still debatable, and cultivation is limited due to the potential ecological risks associated with transgenic plants [10]. Consequently, in view of economical and environmentally friendly management of WBPH, the exploration of host plant resistance approaches has gained tremendous attention. Moreover, various types of dominant and recessive genes for resistance against WBPH have been identified in various rice accessions [11–16]. Complete resistance against the WBPH has not yet been achieved in high-yielding rice cultivars. The management of WBPH using synthetic chemicals has failed because of insecticide resistance [17]. In the current “post-green revolution era,” emphasis is given on sustainability and efficiency rather than on further agricultural intensification, which requires expensive inputs. In pest management, the challenge is to make natural non-chemical methods collectively more effective.

As a major carbon-negative source, Biochar is currently receiving much attention for its amendment in substrate/soil [18]. Biochar addition to the substrate of growing media has been reported to significantly improve plant growth characteristics, mediate heavy metal stress, increase macro- and micronutrients uptake, and improve nutritional quality [19,20]. Kaudal et al. [18] examined the suitability of substituting coir peat with urban biochar in industry-based plant growing media. It was found that volume-based 60% addition was the optimum rate of urban biochar and could improve chemical and physical characteristics in terms of increasing media pH, C:N mass ratio, nutrient content, surface oxidation, and increased air-filled porosity. The amendment also stabilized and increased the bulk density and improved the resistance of particles to breakdown [18]. Similarly, certain biochar additions to sequester carbon from the atmosphere to the soil have been reported to improve the soil tilth, nutrient retention, and bioavailability for plant and crop productivity. Various researchers reported the positive effects of biochar soil amendment on trees and crops grown under controlled and open field conditions [21, 22]. For instance, charcoal-amended soil resulted in greater shoot and root biomass of birch and pine [22]. A single dose of 20 t ha^-1^ of biochar to Columbian savanna soil caused an increase in maize yield by 28–140% when compared to the non-amended control in the second to fourth years after application [21, 23]. These results demonstrate the potential for biochar application to increase plant productivity. However, biochar addition of the same origin shows differential results in substrate and soil. Tender et al. [24] amended soil and substrate with the same type of biochar and observed the effects on lettuce and strawberry. Biochar amendment in the substrate was more pronounced compared to the soil in terms of improving physicochemical properties, fresh and dry biomass, altering rhizosphere microbial community structure, and increasing the foliar resistance against *Botrytis cinerea* [24].

Apart from the physicochemical properties of biochar, its intrinsic nutrient composition plays an important role in enhancing agronomic traits [25]. Biochar with a percentage of C less than 50% and a high amount of macro and micro plant growth elements was categorized as bring nutrient-rich. In this context, mixing nutrient-rich cow manure biochar in sandy soil significantly promoted maize crop parameters, and the effect was attributed to the presence of innate plant nutrients in the manure [26]. To elaborate the effects of two types of biochar with different nutrient constituents, Deenik and Cooney [25] analyzed the performance of nutrient-rich and -poor biochars on maize growth under infertile Oxisol soil. The results revealed that the application of nutrient rich biochar with low C content and high amounts of N, P, S, and ash content significantly promoted maize growth and yield. It has been clearly revealed that overcoming the nutrient deficiency in biochar, often through the addition of N fertilizer, enhances its agronomic efficiency [25, 27, 28]. Chan et al. [27] reported similar findings about the application of green waste biochar that was extremely low in total N and mineral N. Even higher dose of the biochar (100 t ha^-1^) did not show any significant effect on radish yield, until N fertilizer was added.

Although pyrolysis makes biochar sterile, it influences microbial populations and communities and increases beneficial microorganisms [29]. The production of antibiotics and outcompeting pathogens by stimulation of beneficial microorganisms may directly extend protection against soil pathogens [29]. During the addition of biochar to the soil, toxic chemical compounds in the residual tars of biochar may double the killing of soil pathogens [30]. Several antibiotic-producing bacterial strains have been identified in biochar amended soil [30]. The possible role of biochar in inducing plant systemic resistance against pathogenic microorganisms has been evaluated in a number of different systems involving foliar pathogens. For example, Elad et al. [31] found that the biochar amendment significantly reduced disease severity of foliar necrotrophic (*Botrytis cinerea*) and biotrophic (*Oidiopsis sicula*) pathogens in pepper and tomato. Additionally, Harel et al. [32] found that biochar amended substrate suppressed the pathogenicity of *Podosphaera aphanis* powdery mildew, *B*. *cinerea*, and *Colletotrichum acutatum* on strawberry leaves. Together these findings suggest that biochar could induce a systemic response in plants against pathogens. There was no direct toxicity towards the pathogen, as the location of biochar was spatially separated from the site of infection during all stages of plant development [32].

Plants protect themselves from biotic stresses, such as damage from necrotrophic pathogens and leaf-chewing herbivores [33, 34]. Jasmonic acid (JA) is among the major phytohormones during biotic stress resistance, e.g., it releases volatiles to indirectly kill herbivores by attracting its natural enemies or directly by producing toxic compounds to deter invaders [33]. Jasmonic acid also plays a role in the regulation of important developmental processes (fertility, tendril coiling, sex determination, and leaf senescence) [35]. Jasmonic acid and its derivatives are derived from linolenic acid, and their biosynthesis takes place in the chloroplasts and peroxisomes, respectively [35]. Despite studies on the role of jasmonic acid in rice defense responses can accumulate plant volatile compounds has been conducted [35]. Still, in-depth investigations are necessary to gain a more comprehensive understanding of jasmonic acid regulation as a mechanism in rice grown under various conditions for handling biotic stress in order to improve important agricultural traits.

The present study was conducted to evaluate the optimum biochar level for plant growth promotion in Japonica rice cultivars with varying levels of resistance to WBPH. A supplementary experiment was designed to test the effect of substrate amended with the optimized biochar level on the resistance and susceptibility of rice cultivars and its role in the enhancement of induced systemic resistance with or without WBPH infestation by measuring levels of endogenous jasmonic acid content. It is obvious that jasmonic acid induces defense, but it is unknown whether this is at the expense of growth suppression by delaying cell division/growth and depleting energy reservoirs or simply to promote one trait by scarifying the other [36]. Therefore, quantification of endogenous jasmonic acid was intended to analyze the extent to which growth will be affected in case of induced systemic resistance offered by biochar application.

## Materials and methods

### Substrate preparation and addition of different biochar doses for initial screening

In this study, the substrate TBT (Soil and Fertilizer Technology, Korea) was used. The nutrient composition of the substrate consisted of peat moss (10–15%), perlite (35–40%), coco peat (45–50%), and zeolite (6–8%); it contained NH_4_^+^ (~0.09 mg g^−1^), NO_3_^−^ (~0.205 mg g^−1^), P_2_O_5_ (~0.35 mg g^−1^), and K_2_O (~0.1 mg g^−1^). The biochar was obtained commercially from Kangwon Grasses Industries Ltd. (Kangwondo, South Korea). Biochar consists of deciduous trees (70%), dolomite (20%), and molasses (10%), is derived via slow pyrolysis, and is alkaline in nature. Biochar has an average particle size of ≤ 5 mm, a moisture level of 7.4%, and an ash content of 2–3%. ICP-OES analysis confirmed the presence of 24.55% C, 1.154% N, and with the remaining contents measured in mg kg^-1^: 27, Al; 124841, Ca; 0.16, Cu; 1774, Fe; 12009, K; 61272, Mg; 9, Mn; 2252, Na; 0.05, Zn; and 440, P. Mb, B, Ni, and Co were not detected at a 0.01 mg kg^-1^ limit of detection. Biochar concentrations of 1%, 2%, 3%, 5%, 10%, 15%, and 20% were added to the substrate based on weight and mixed well by stirring and rotating end-over-end in sealed plastic bags to incorporate it homogeneously. Substrate not containing biochar served as a control. Prior to the addition of water, all substrate samples were moistened to half of their dry weight and left for 7 d in dark conditions at room temperature to equilibrate. The plastic pots (25 cm × 20 cm × 20 cm) filled with 1 kg of substrates and 3 L of double distilled water (DDW) with and without designated rates of biochar doses were then autoclaved (at 121°C for 15 min) three times to create microbe-free conditions for semi hydroponic media. Three pots per treatment prepared using a similar method were used throughout the experiments.

### Experimental setup 1

#### Plant material and rice growth response to different biochar doses for initial screening under controlled chamber conditions

Rice (*Oryza sativa* L.) seeds of two cultivars ‘Nagdong’ and ‘Cheongcheong’ were obtained from the Rice Genetic Resource Center (Kyungpook National University, Daegu, South Korea). Recently, Yun et al. [37] reported Cheongcheong as moderately resistant and determined that Nagdong has both a positive and negative resistance against WBPH [37, 38]. The Cheongcheong seeds and Nagdong seeds used in this experiment are the F2 cross of Cheongcheong and Nagdong (see Yun et al. [37], Kim et al. [38], and Vicheka et al. [39]). Due to segregation, both the susceptibility as well as resistance are expected at this stage. Meanwhile, the F2 material was considered good enough for WBPH experiment because it is the segregation seeds after large produced double haploid lines. This is the reason we chose these varieties to determine and easily identify the resistance of their ‘Cheongcheong/Nagdong’ double haploid (CNDH) population against WBPH. The genealogical population was the development of Cheongcheong/Nagdong Doubled Haploid (CNDH). The population cross of YR675-153-2-2/IR2035-290-2 resulted in Cheongcheong (Milyang46), and that of Nonglim No.6/Mineyyudaka resulted in Nagdong (Milyang15). The procedure entailed the continuous crossing of Cheongcheong (Milyang46)/ Nagdong (Milyang15), which yielded Cheongcheong/Nagdong. We used for constructing a genetic map, and we developed Cheongcheong/Nagdong through another culture of the F1 derived from a cross between Cheongcheong and Nagdong, ultimately resulting in Cheongcheong/Nagdong Doubled Haploid (CNDH). F2 segregation is good for the vertical generation in rice transplanting. Milyang 46 and Milyang 15 are the systematic numbers named in the regional adaptability experiment after completion of the yield tester. These varieties were named 'Cheongcheong' and 'Nagdong', respectively, to register the brand name. The seeds were healthy, with 6% moisture content and 95% germination rate. At the same time as preparing the pots, the seeds were germinated (28°C and relative humidity of 60%) for 10 d to obtain seedlings of similar size in the germination trays. Prior to germination, the seeds were surface sterilized in autoclave pots with 2.5% sodium hypochlorite for 30 min and were then rinsed with autoclaved DDW. After germination, seedlings of similar size were randomly selected, and transplanted to their respective plastic pots that were prepared earlier.

The plastic pots were fitted with Styrofoam sheets. Fifteen holes of equal size at equal distance were made in Styrofoam sheets, and foam was used to fill the gap and support the rice seedlings. The semi-hydroponic media were aerated daily for 2 h with the help of air pumps to avoid harmful decreases in oxygen levels, and water was added to the pots to maintain sufficient moisture levels. A total of 16 treatments were completed in the initial screening, for which the Nagdong and Cheongcheong rice cultivars were grown in the control substrate (biochar 0%), and substrates amended with 1%, 2%, 3%, 5%, 10%, 15%, and 20% biochar in pots for 15 d. After 15 d of growth in a controlled growth chamber (KGC-175 VH, KOENCON, South Korea) with regulated conditions (14-h light, 06:00–20:00 h, 30°C, relative humidity 70% and 10 h dark, 20:00–06:00 h, 25°C, relative humidity 70%), growth parameters (shoot/root length, chlorophyll content) were recorded. The total chlorophyll content of the second-most upper leaves were determined using a chlorophyll meter (Soil-Plant Analysis Development (SPAD-502); Minolta, Japan). Plants were harvested and immediately stored in liquid nitrogen. For dry weight, five plants were randomly selected from each replication in all treatments and oven dried at 70°C for 72 h. The weights of oven-dried plants were measured using a sensitive electronic scale.

### Experimental setup 2

#### Plant growth response in optimized biochar substrate under WBPH attack

Using the results from the initial screening (experimental setup 1), we selected two weight-based biochar doses that promoted maximum plant growth (biochar 10% equivalent to 27 t ha^-1^ by using layer of substrate = 0.1 m and its bulk density = 0.3 Mg m^-3^) and inhibition (biochar 20%) along with the control (biochar 0%) for the second experiment. The methods for rice seed germination, seedling selection, transplantation, and closely regulated growth conditions for 15 d were the same as previously described for the initial screening. The plants were then divided into two groups, one of which was subjected to WBPH and the other not. The entire experimental setup was then shifted to a greenhouse with environmental conditions identical to that of the growth chamber. Both WBPH and no-WBPH sample groups were placed in boxes (1 m × 1 m × 1 m) covered with a nylon screen mesh. The purpose of the mesh was to prevent the escape of WBPH and infestation of the no-WBPH control group. For the WBPH group, 10 insects (see details below for rearing and domestication) in their second to third instars were applied to each plant. After infestation, WBPH were monitored daily and were generally observed on the rice stem or leaf sheaths and partly on leaf surfaces. The difference in plant growth parameters among the three treatments may cause the variation in the distribution of WBPH. Therefore, the numbers of WBPH were regularly counted and found to be the same in each plant to rule out a numbers effect. Throughout the course of biotic stress, WBPH were not seen resting on the mesh wall of the cages. The experiment included the following treatments: (1) Cheongcheong rice without biochar, (2) Cheongcheong rice with 10% biochar, (3) Cheongcheong rice with 20% biochar, (4) Cheongcheong rice with WBPH and without biochar, (5) Cheongcheong rice with 10% biochar and WBPH, (6) Cheongcheong rice with 20% biochar and WBPH, (7) Nagdong rice without biochar, (8) Nagdong rice with 10% biochar, (9) Nagdong rice with 20% biochar, (10) Nagdong rice with WBPH and without biochar, (11) Nagdong rice with 10% biochar and WBPH, and (12) Nagdong rice with 20% biochar and WBPH. The rice plants were left to grow for 6 d with stress application (WBPH infestation) under controlled greenhouse conditions (day/night cycle: 14 h at 30°C/10 h at 25°C; relative humidity 60–70%). The semi-hydroponic media were aerated daily for 2 h, and tap water was added to maintain moisture levels. The plant’s growth parameters including their resistance score were recorded, and the plants were then immediately stored in liquid nitrogen after harvesting and were freeze-dried for 1 wk. (VirTis Freeze Dryer, Gardiner, NY, USA). The Standard Evaluation System for Rice (IRRI) detail given in the footnote of Table 1 was used for scoring resistance to insect infestation (IRRI, 1988).

**Table 1:** Disease scoring of Cheongcheong and Nagdong rice varieties in the presence and absence of biochar (BC).

Treatment	Damage Score
**Cheongcheong with WBPH infestation**	
**Con + WBPH**	6.20±1.26^b^
**BC 10% + WBPH**	8.47±1.41^a^
**BC 20% + WBPH**	9.00±0.00^a^
**Nagdong with WBPH infestation**	
**Con + WBPH**	5.00±0.00^a^
**BC 10% + WBPH**	3.13±0.52^b^
**BC 20% + WBPH**	5.27±1.98^a^

Scale: 0 = No damage, high resistance; 1 = very slight damage, resistance; 3 = first and second leaves with orange tips, slight stunting, moderate resistance; 5 = More than half the leaves with yellow-orange tips, stunting, moderate susceptibility; 7 = more than half of plants dead, remaining plants severely stunted and wilted, susceptible; 9 = All plants dead, high susceptibility.

WBPH = White-backed plant hopper; Con = potting media of both Cheongcheong and Nagdong rice varieties without any treatment; BC 10% = application of biochar at rate of 10% (w/w) to the potting media of both Cheongcheong and Nagdong rice varieties; BC 20% = application of biochar at rate of 20% (w/w) to the potting media of both Cheongcheong and Nagdong rice varieties.

#### Domestication and bioassay of WBPH

The WBPH population was obtained from the National Institute of Crop Sciences (NICS), Rural Development Administration (RDA), South Korea and reared inside an insect room according to the methods described by Yun et al. [37] and Vicheka et al. [39]. The temperature and day and night conditions for insect rearing were set to 27°C, 16 h, and 8 h, respectively. The insects were continuously fed with freshly grown WBPH-susceptible japonica rice seedlings ‘Chucheong,’ which were changed twice weekly. Oviferous hoppers were separated and maintained in special screened cages (50 cm × 50 cm × 40 cm) to obtain second and third stage instar nymphs that could be used for infesting seedlings in the relevant sample groups.

#### Endogenous jasmonic acid analysis

Endogenous jasmonic acid was extracted from seedlings according to the protocol in McCloud and Baldwin [40]. The extracts were then analyzed by GC/MS–SIM (6,890 N network GC system and 5,973 network mass selective detector; Agilent Technologies). To enhance the sensitivity of the method, spectra were recorded in the selected ion mode, i.e., to determine jasmonic acid content, we monitored the fragment ion at *m/z* = 83 amu, corresponding to the base peaks of JA and [9, 10-^2^H_2_]-9, 10-dihydro-JA. The amounts of endogenous jasmonic acid were calculated from the peak areas of jasmonic acid compared with the corresponding standards.

### Statistical analysis

Experiments 1 and 2 were repeated three times in a completely randomized design, and each treatment was replicated six times. ANOVA was employed for statistical analysis, and the mean values were compared with the Duncan multiple range test (DMRT) (*P* < 0.05) using the statistical software program SAS (version 9.2, Cary, NC, USA).

## Results and discussion

### Experiment 1

#### Initial screening of rice cultivars for the determination of optimum biochar dose

The results revealed that the biochar amendment at various concentrations showed differential effects on plant growth parameters such as shoot/root length, fresh/dry weight of root/shoot, and chlorophyll content (Table 2). The addition of biochar at different concentrations resulted in a very unique trend. Biochar at lower concentrations (e.g., 1%, 2%, and 3%) negatively affected growth, while a 5% biochar concentration showed a stimulatory effect. However, in both Cheongcheong and Nagdong varieties, the greatest effect was observed at a concentration of 10% biochar. This concentration significantly (*P* < 0.05) increased all plant growth parameters compared with the control. A decline for all growth parameters was observed at 15% biochar and 20% biochar. However, the 20% biochar presented significant inhibitory effects on plant growth compared with 10% biochar and the control. Therefore, all further experiments were executed with the optimal biochar concentration of 10% along with plants with and without biochar concentrations of 20% as a control. Furthermore, with and without biochar application, plant growth parameters of Cheongcheong and Nagdong were significantly different (*P* < 0.05) from each other.

**Table 2:** Initial screening results for the determination of optimized biochar application rate as revealed by plant growth parameters in Cheongcheong and Nagdong rice varieties.

Treatment	SL (cm)	RL (cm)	FW (gm)	DW (gm)	CC (SPAD)
**Cheongcheong**					
**Control**	20.44± 8.95^bc^	12.00±3.24^bc^	11.88±0.093^d^	1.68±0.0035^d^	21.38±2.46^b^
**BC 1%**	15.11±2.64^cd^	11.56±2.70^c^	6.95±0.046^f^	0.87±0.0086^g^	14.66±8.80^b^
**BC 2%**	14.94±2.84^cd^	10.61±2.69^c^	5.55±0.037^g^	0.95±0.0008^f^	11.79±6.02^bc^
**BC 3%**	13.11± 4.48^d^	9.56± 4.85^c^	4.42±0.013^h^	0.71±0.0053^h^	17.22±8.00^c^
**BC 5%**	27.00±1.73^b^	15.57±6.96^ab^	17.61±0.024^b^	2.24±0.0053^b^	21.71±1.39^b^
**BC 10%**	32.00±3.97^a^	16.39±2.52^a^	19.76±0.086^a^	2.47±0.0073^a^	26.60±1.20^a^
**BC 15%**	25.37±2.63^b^	13.11±0.93^abc^	13.45±0.047^c^	1.76±0.0015^c^	19.62±9.68^bc^
**BC 20%**	19.83±2.78^bc^	11.67±1.12^c^	9.68±0.072^e^	1.61±0.0013^e^	12.84±6.25^bc^
**Nagdong**					
**Control**	34.50±1.71^c^	13.33±1.87^b^	19.89±0.065^c^	2.05±0.0094^d^	15.42±7.84^c^
**BC 1%**	23.44±6.40^d^	14.78±3.19^b^	15.82±0.046^d^	1.99±0.0097^e^	18.67± 10.06^bc^
**BC 2%**	23.61±2.29^d^	10.00±2.12^c^	10.94±0.038^e^	1.51±0.0009^g^	14.89± 8.00^c^
**BC 3%**	20.33±6.41^d^	8.94±1.93^c^	7.03±0.09^f^	1.20±0.0088^h^	14.91±6.10^c^
**BC 5%**	36.39±3.97^c^	19.22±2.33^a^	16.9±0.035^d^	2.38±0.0079^c^	26.29± 2.01^b^
**BC 10%**	45.22±2.31^a^	20.12±5.73^a^	32.41±0.011^a^	5.24±0.0019^a^	31.00± 3.49^a^
**BC 15%**	41.14±1.62^b^	13.81±0.86^b^	29.87±0.034^b^	3.39±0.0046^b^	25.36±1.58^b^
**BC 20%**	25.75±10.63^d^	9.44±0.73^c^	18.70±0.025^c^	1.85±0.0086^f^	12.51±8.10^c^

SL = Shoot Length; RL = Root Length; FW = Fresh Weight; DW = Dry Weight; C.C = Chlorophyll Content; SPAD = Soil-Plant Analysis Development.

Control = potting media of both Cheongcheong and Nagdong rice varieties without any treatment; BC 1%, 2%, 3%, 5%, 10%, 15%, and 20% = application of biochar at rate of 1%, 2%, 3%, 5%, 10%, 15% and 20% (w/w) to the potting media of both Cheongcheong and Nagdong rice varieties, respectively.

Mean values ± standard error (n = 27) represent the analyzed data pooled from three independent consecutively conducted experiments. Values in a column accompanied with different superscripted letters are significantly different at *P* < 0.05 as indicated by DMRT.

It is obvious that the plant growth parameters decreased as the biochar dose increased from 1%–3%, while the growth attributes suddenly increased at 5% biochar. It is known that biochar can produce bi-phasic plant responses; at low to optimum concentrations, it often promotes plant growth or defenses to pests and pathogens and vice versa at high concentrations. However, here the addition of 1%–3% biochar caused a surprising effect and might have shifted the bi-phasic plant response to the negative side along the biochar concentration gradient. Our results were consistent with those of Kammann et al. [41], who suggested that the negative effect on growth parameters would have been primarily caused by the capture of nitrate and other nutrients. Several other authors also observed that biochar captured nitrate and other nutrients [42–44].

Although we have identified an optimal concentration for biochar amended substrate/growth medium (10%) in our experimental setup, there is currently no standard recommended application dosage for biochar. Dosages for biochar can depend on several factors and need to be determined separately for the specific purposes of the application, whether in an agronomic or environmental context [45, 46]. A number of authors have identified several factors that need to be investigated in different scenarios, including variation in the physical and chemical properties of biochar, substrate, and differences in the responses of different plant species [24, 32, 47, 48]. Several studies have identified the beneficial effects and optimum rates of biochar amendment in different types of substrate [18, 19, 20, 24, 30, 32, 45, 46, 48]. The previous studies of Méndez et al. [49, 50] and Nieto et al. [51] reported the promising effect of biochar mixing with peat substrate at higher rates, such as 10%, 50% or 75% v/v, to find a low-cost solution for growing horticultural crops. The experiment conducted by Méndez et al. [50] demonstrated that the addition of biochar (10% volume rate) to peat increased plant biomass and shoot length of lettuce plants by 184–270% and 137–147%, respectively. Steiner and Harttung [48] compared the effect of biochar amendment in different types of growing media and found that biochar addition up to 75% in peat could replace lime for enhanced plant growth by favorably increasing pH and lowering electrical conductivity. Ruqin et al. [45] reported that substrate amendment with an optimum rate of biochar (100 mL L^-1^) and super absorbent polymer (0.8 g L^-1^) improved physical and chemical properties of spent pig litter compost substrate, which subsequently increased plant growth and macro- and micronutrient uptake of water spinach under greenhouse conditions.

In a similar context, authors Mia et al. [47] analyzed the effects of biochar at different applications (0, 10, 50, and 120 t ha^-1^) on biologically driven N fixation and total biomass in common garden experiments. Mia et al. [47] found that a biochar application at the rate of 10 t ha^-1^ to the soil significantly increased N fixation and total biomass in their study species: red clover grown alone or in combination with red fescue grass and plantain. Furthermore, the results obtained at 120 t ha^-1^ of biochar showed a negative impact on the biological parameters and, hence, corroborate our findings of rice growth retardation at biochar 20%. Liu et al. [52] applied biochar at two different rates (8 and 16 g kg^-1^) to ryegrass grown under water-limited conditions in separate treatments of low and adequate fertile soil. The application of different biochar doses presented variable outcomes, and the 8 g kg^-1^ of biochar was shown to be the optimum dose by resulting in improved plant height and biomass in low fertile soil, compared to biochar applied at 16 g kg^-1^ as well as the control. Pratiwi and Shinogi [53] applied two doses of rice husk biochar (2% and 4%) to Japonica rice grown in pots subjected to field conditions. Among the growth parameters, stem height showed a significant positive response to biochar 4%, while root length and other plant growth parameters were only marginally increased. In another greenhouse pot study, the application of biochar derived from peanut shell at the concentration of 6% in red-ferrosol and redoxi-hydrosol soil improved several biomass parameters (leaf, stem, root, and pod) in commercial peanut plants compared with biochar concentrations of 0.375%, 0.750%, 1.50%, 3.00%, and the control [54]. A two-year field study conducted by Dong et al. [55] revealed that rice straw-derived biochar at a dose of 22.5 t ha^−1^ resulted in higher rice yield and longer stem length. Furthermore, a study accounting for the potential conditions under future climate change—such as higher CO_2_ levels and temperatures—demonstrated that biochar application improved both the total and aboveground biomass under predicted and normal conditions [56]. In a study conducted by Yeboah et al. [57], biochar at two rates (2.5 t ha^-1^, 5 t ha^-1^) was combined with three different NPK fertilizer doses, with the purpose of minimizing the use of inorganic fertilizers on maize grown in small fields. Among the treatments, the biochar application at a rate of 5 t ha^-1^ was shown to be the most effective treatment. However, the authors also recommended that, in cases where feedstock availability was limited, the application of biochar at the rate of 2.5 t ha^-1^ combined with half of the NPK dose could also produce an optimum yield. In summary, each of these studies signifies the importance of optimizing the correct dose of biochar in order to produce maximum yields in an economical way. They also highlight the importance of employing the appropriate experimental processes for the agronomic or environmental context before recommending an optimum dose.

The most likely explanation for the results of the current study and previous studies is that biochar amendment provides a lasting supply of additional nutrients, as well as improves nutrient retention and the water holding capacity of the soil. In the present study, it was also observed that the biochar used contained a variety of macro- and micronutrients. One of the most important factors that specify the usage of a particular type of biochar is its intrinsic nutrient composition (rich or poor), which could produce varying results in the form of significant differences in plant growth, soil properties and nutrient accumulation in crop plants [25, 58]. Abbasi and Anwar [58] found that biochar obtained from two different sources—white clover residues and poultry manure—respectively showed a differential response upon application in maize and wheat. Overall, poultry manure biochar enhanced plant growth characteristics more than from white clover residues. The relatively lower C, narrower C:N ratio, higher total N, and ash content in poultry manure biochar was attributed as the apparent explanation for these promising results. The study of Yue et al. [59] demonstrated that nutrient rich biochar could potentially influence the soil fertility status in a short period of time. Furthermore, Yue et al. [59] found that the application of municipal sewage sludge-derived biochar to the poor urban soil acted as a soil conditioner and significantly stimulated the growth and dry weight of turf grass by enhancing plant mineral nutrition. In another study, Khan et al. [28] investigated the effect of nutrient rich sewage sludge biochar on rice growth parameters and soil fertility. The sewage sludge biochar improved grain yield, biomass, and the accumulation of P and Na in tissues. Further beneficial effects were observed on soil physical and chemical properties like pH, nutrient availability, soil organic carbon, and total N, in addition to decreasing some of the hazardous heavy metals. All of these reasons justify the addition of molasses and dolomite to the hardwood biochar used in our study, thus making it nutrient rich and capable of dual-purpose use, i.e., carbon sequestration and agronomic potential in the form of fertilizer.

### Experiment 2

#### Plant growth attributes with or without WBPH attack under optimum biochar application

**Fig 1:**
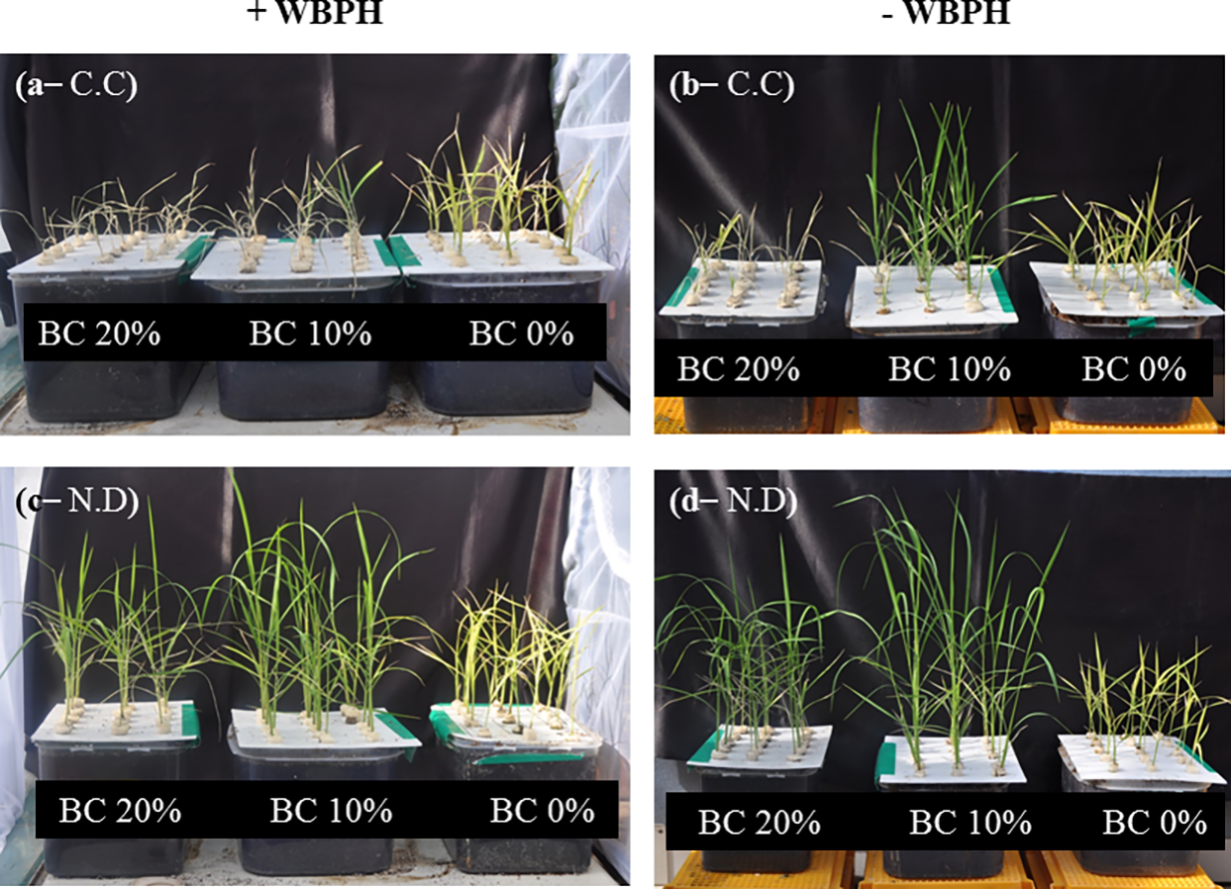
**Cheongcheong–CC (a, b) and Nagdong–ND (c, d) rice varieties in the presence or absence of biochar with (+) or without (-) WBPH infestation.** For the evaluation of biochar effect on the resistance and susceptibility of rice, ten insects of WBPH were applied to each designated plant for 6 d under control condition. From right to left in Figs a, b, c and d, Control (BC 0%) = plants without any treatment; i.e., no biochar application in both CC and ND rice varieties; BC 10% = application of biochar at rate of 10% (w/w) to the potting media of both CC and ND rice varieties; BC 20% = application of biochar at rate of 20% (w/w) to the potting media of both CC and ND rice varieties. Figs (a, b, c, and d) represents the effect of treatments from three independent consecutively conducted experiments.

When comparing the Cheongcheong and Nagdong rice varieties with and without WBPH infestation, and in the presence and absence of biochar, significant differences were observed in the plant growth parameters (Table 3; Fig 1). In Cheongcheong, the sample group subjected to WBPH infestation had no significant differences in the plant parameters between 10% biochar and the control, with the exception of chlorophyll content being higher in 10% biochar. However, 20% biochar demonstrated a significant reduction in the growth parameters compared with 10% biochar and the control. In the Nagdong sample group subjected to WBPH infestation, a biochar concentration of 10% resulted in significant increases in the plant parameters (shoot length, fresh weight, dry weight, and chlorophyll content) (*P* < 0.05) compared with 20% biochar and the control. As with the initial screening experiment, for the sample groups (both Cheongcheong and Nagdong) not subjected to WBPH infestation, a biochar concentration of 10% showed a significant (*P* < 0.05) promoting effect on the analyzed growth parameters.

**Table 3:** Cheongcheong and Nagdong rice varieties growth response in the presence or absence of biochar (BC) with or without WBPH attack.

Treatment	SL (cm)	RL (cm)	FW (gm)	DW (gm)	CC (SPAD)
**Cheongcheong without and with WBPH attack**					
**Con**	15.43±2.12^b^	7.07±1.03^b^	5.64±0.12^b^	0.97±0.042^b^	15.39±2.64^b^
**BC 10%**	23.17±3.77^a^	9.57±1.08^a^	8.14±0.24^a^	1.34±0.039^a^	26.43±4.63^a^
**BC 20%**	11.97±1.73^c^	5.70±1.49^c^	3.65±0.16^c^	0.56±0.045^c^	13.58±2.62^b^
**Con + WBPH**	14.60±3.29^a^	9.13±2.23^a^	6.62±0.31^a^	0.95±0.025^a^	10.07±3.60^b^
**BC 10% + WBPH**	16.47±2.18^a^	8.85±2.67^a^	6.33±0.24^a^	0.90±0.044^a^	16.43±2.69^a^
**BC 20% + WBPH**	11.20±3.08^b^	6.47±1.77^b^	3.62±0.21^b^	0.57±0.031^b^	7.59±0.82^c^
**Nagdong without and with WBPH attack**					
**Con**	35.30±3.14^b^	6.57±0.46^a^	8.75±0.40^b^	1.58±0.079^b^	22.87±2.63^b^
**BC 10%**	45.87±3.54^a^	7.07±0.42^a^	18.25±0.22^a^	2.46±0.035^a^	31.23±5.83^a^
**BC 20%**	20.33±3.26^c^	5.43±1.57^b^	7.60±0.18^c^	1.22±0.017^c^	17.94±3.49^c^
**Con + WBPH**	25.20±3.76^b^	7.33±0.90^a^	10.96±0.19^b^	1.29±0.08^b^	22.03±2.72^b^
**BC 10% + WBPH**	32.53±3.87^a^	7.53±0.83^a^	15.19±0.36^a^	2.14±0.023^a^	29.86±2.98^a^
**BC 20% + WBPH**	20.33±3.18^c^	6.27±0.72^b^	8.41±0.11^c^	1.26±0.01^c^	18.67±2.62^c^

SL = Shoot Length, RL = Root Length, FW = Fresh Weight, DW = Dry Weight, CC = Chlorophyll Content, SPAD = Soil-Plant Analysis Development.

WBPH = White-backed plant hopper; Con = potting media of both Cheongcheong and Nagdong rice varieties without any treatment; BC 10% = application of biochar at rate of 10% (w/w) to the potting media of both Cheongcheong and Nagdong rice varieties; BC 20% = application of biochar at rate of 20% (w/w) to the potting media of both Cheongcheong and Nagdong rice varieties.

Mean values ± standard error (n = 27) represent the analyzed data pooled from three independent consecutively conducted experiments. Values in a column accompanied with different superscripted letters are significantly different at *P* < 0.05 as indicated by DMRT.

WBPH infestation in the control plants (without biochar) significantly reduced shoot length in Nagdong and the chlorophyll contents of Cheongcheong, compared with their respective controls that were not subjected to WBPH infestation. This could likely be explained by previous research which has demonstrated that WBPH infestation reduces plant vigor and growth, induces leaf discoloration, and delays tillering emergence, grain setting, and dehydration [60–62]. Furthermore, WBPH are highly problematic for the cultivation of Chinese hybrid rice varieties. Infestation not only mechanically damages the rice plants but also transmits viruses which can hasten the onset of symptoms, as well as increase the magnitude of existing stresses. The devastating impacts that WBPH can cause have made it one of the most important pests of rice in Asia, and it is responsible for severe losses in rice production [61].

#### Evaluating the effects of biochar on the resistance of rice varieties to WBPH

The Nagdong and Cheongcheong varieties were evaluated for their resistance to WBPH with and without biochar amendment (Table 1, Fig 1). The control Cheongcheong (Con + WBPH) showed some damage with a resistance rating of 6.20 ± 1.26, with more than half of plant leaves remaining yellow, stunted, and wilted, whereas the control Nagdong (Con + WBPH), was rated 5.00 ± 0.00. Interestingly, the biochar amendment resulted in significantly different effects on each variety. In the case of Cheongcheong, 10% biochar and 20% biochar applications significantly decreased the WBPH resistance, resulting in resistance scores of 8.47 ± 1.141 and 9.00 ± 0.00, respectively. Conversely, Nagdong resistance was significantly increased with additions of 10% biochar and 20% biochar, with resistance ratings of 3.13 ± 0.52 and 5.27 ± 1.98, respectively.

In this study, the first and foremost issue that was considered concerns the difference in the environmental conditions of WBPH rearing and plant growth conditions that might affect the results. However, no such effect was noted that could bias our results. Previous studies that did not report an effect should not imply the absence of an effect. We all know that circadian rhythm would affect behavior. Here it is noted that the score for resistance and susceptibly were made on a visual observation basis where chance of error cannot be denied. Furthermore, Cheongcheong is the result of a Japonica and Indica rice cross and shares 70–80% and 30–20% genes from both varieties, respectively. Yun et al. [37] determined that segregated populations derived from the cross of the same Japonica and Indica varieties revealed higher frequencies of the Cheongcheong allele than that of the Nagdong allele, and they later act as a donor for increasing allele.

Only a few studies have addressed the potential of biochar soil amendment to alter plant resistance to pathogens and herbivorous insects. For example, biochar pyrolyzed from eucalyptus woodchips and pepper plant waste products was shown to decrease the severity of *R*. *solani* infection in beans [63]. In another related study, Harel et al. [32] applied biochar derived from pepper plant waste products to strawberry plants under different infection treatments and found that it suppressed three foliar diseases caused by *Botrytis cinerea*, *Colletotrichum acutatum*, and *Podosphaera aphanis*. Additionally, the application of biochar pyrolyzed from citrus wood to tomato plants suppressed the infection of *B*. *cinerea* and *Leveillula taurica*—pathogens responsible for causing gray mold and powdery mildew [31]. Lehmann et al. [64] have suggested that the potential mechanisms by which biochar induces systemic plant defenses against pathogens and promotes growth may include enhanced nutrient solubilization and uptake.

Due to the significant threat that WBPH infestations pose to rice crops, considerable effort has been invested in discovering a genetic solution to resistance. For example, breeding programs have so far identified 14 resistance-enhancing genes and incorporated them in to existing rice varieties [38]. Moreover, the relevant quantitative trait loci in all 12 rice chromosomes have been identified to add further genetic resolution that can be used to strengthen the breeding program. In our study, biochar amendment resulted in significant and interesting effects on the resistance of the Cheongcheong and Nagdong rice varieties. Our evaluation resulted in contrasting results between the two varieties, with Nagdong gaining increased resistance, whereas Cheongcheong decreased in resistance to WBPH infestation. To the best of our knowledge, this is the first study demonstrating that biochar amendment has contrasting effects on the resistance levels towards WBPH of two different rice varieties. However, our results do corroborate the findings of Kajimura et al. [65] regarding the difference in resistance of the susceptible variety between the control and 10% biochar-treated plants. In their field experiment, Kajimura et al. [65] found that biochar amendment significantly lowered the WBPH population density compared to treatment with a synthetic fertilizer. Regarding our results with the Cheongcheong variety, previous research suggests that the application of biochar may have altered the metabolism of plants and subsequently changed the nature of plant volatiles. Plant volatiles have been proven to play an important role in the susceptibility of rice varieties to WBPH infestation. For example, Khan and Saxena [62] analyzed the effects of volatiles in the form of steam distillates derived from resistant and susceptible rice on WBPH and found that the odors of volatiles of the resistant variety repelled WBPH, whereas odors of the susceptible variety attracted WBPH. The same study also demonstrated that the treatment of susceptible rice with plant volatile extracts of resistant rice reduced the feeding behavior or metabolism of WBPH [62]. Previous studies have suggested that the addition of P and N at higher levels than normal can significantly enhance the male and female longevity of WBPH in both resistant and susceptible rice [66]. Therefore, in the present study, the additive effect of biochar on available nutrients, consistent with that reported by Waqas et al. [20], could have likely resulted in such changes occurring only in the Cheongcheong variety. The difference between our results and those of Salim and Saxena [66] regarding the increased resistance of the Nagdong rice could simply be attributed to the vigorous physiological response of the Nagdong variety compared to that of the Cheongcheong variety. This may have also made the nutrient status of the Nagdong variety unfavorable to WBPH due to the dilution of nutrients resulting from more plant mass per unit of nutrient, and the opposite effect in the Cheongcheong variety.

#### Contrasting effects of biochar on jasmonic acid contents versus rice growth and WBPH resistance in Cheongcheong and Nagdong varieties

**Fig 2:**
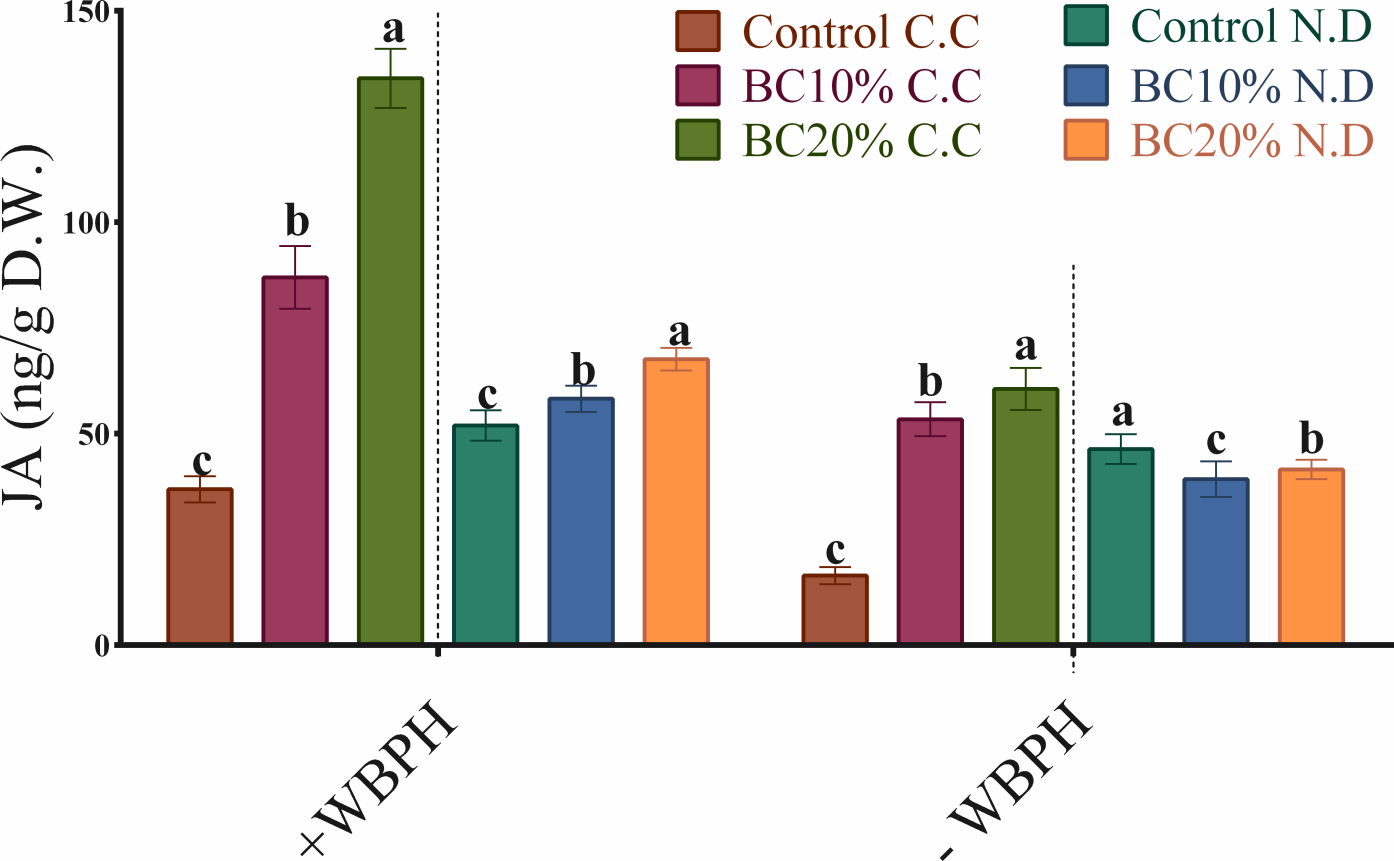
Regulation of jasmonic acid (JA) content in Cheongcheong–CC and Nagdong–ND rice varieties in the presence or absence of biochar with (+) or without (-) WBPH infestation. JA levels in rice shoots under all mentioned treatment circumstances were measured to understand the effect of biochar on induced systemic resistance under WBPH infestation. Control = plants without any treatment; i.e., no biochar application in both CC and ND rice varieties; BC 10% = application of biochar at rate of 10% (w/w) to the potting media of both CC and ND rice varieties; BC 20% = application of biochar at rate of 20% (w/w) to the potting media of both CC and ND rice varieties. Mean values ± standard error (n = 3) in the form of column bars and error bars denote the analyzed data pooled from three independent consecutively conducted experiments. Different lowercase letters on each column shows the significant difference (*P* < 0.05) among different treatments in each group as indicated by DMRT.

The addition of biochar showed contrasting effects on jasmonic acid contents versus significant growth promotion of Cheongcheong and Nagdong under no WBPH condition (Fig 2, Table 3). The application of biochar to Cheongcheong significantly increased its jasmonic acid content in the absence of WBPH infestation (Fig 2). In the absence of WBPH, jasmonic acid content increased by three-fold and four-fold under 10% and 20% biochar, respectively, compared to the Cheongcheong control. In contrast, the application of biochar to Nagdong significantly reduced the jasmonic acid content in the absence of WBPH.

The same pattern of increased jasmonic acid content was observed in Cheongcheong in the presence of WBPH infestation under 10% biochar and 20% biochar compared with the Cheongcheong control. Regarding Nagdong, 20% biochar significantly increased jasmonic acid content, with 10% biochar having a less significant increasing effect, compared with the Nagdong control.

Biochar amendment has been shown to increase exogenous ethylene (ET) production and alter plant metabolic activities, thereby inducing resistance to pathogens [64, 67]. Hence, it could be assumed that exogenous ET interacts with jasmonic acid and other green volatiles to promote induced systemic response pathways in plants. The same function of ET was previously found in the activation of the jasmonic acid dependent defense against root knot nematodes. Huang et al. [68] suggested that biochar amendment induced priming in rice partly via an ET signaling pathway, thereby reducing infestation of root knot nematodes. Regarding our results of jasmonic acid contents increasing in Cheongcheong in the absence of WBPH infestation (Fig 2), this could be explained by biochar amendment producing the priming effect suggested by Huang et al. [68]. Looking at these results that increase in jasmonic acid level parallel to plant growth promotion under 10% biochar in the absence of WBPH is contradictory to common jasmonic acid behavior. However, it can be inferred that the same jasmonic acid level may be optimum for growth promotion in Cheongcheong. This result is also corroborated by the findings of Waqas et al. [19], wherein the priming effect caused by biochar application increased jasmonic acid levels in soybean plants under normal conditions. However, when exposed to heavy metals, the same primed soybean plants exhibited stress amelioration by lowering their endogenous jasmonic acid levels. In contrast, in our results this priming effect was followed by an apparent increase rather than a decrease in the biosynthesis of jasmonic acid in response to WBPH infestation [69]. This may correspond to an overproduction of jasmonic acid in stressful conditions, thereby negatively effecting plant growth and plant resistance. Machado et al. [36] recently reported same findings that simulated herbivore attack of *Manduca sexta* in *Nicotiana attenuata* increased jasmonates contents therefore significantly reduced growth by impairing secondary metabolites, sugar contents and antagonizing gibberellins signaling. Contrastingly, Nagdong lowered the jasmonic acid level under biochar application without WBPH attack and promoted plant growth, which is a result consistent with the findings of Viger et al. [70]. However, as with Cheongcheong, jasmonic acid levels in Nagdong also increased under biotic stress. To improve our understanding of these reactions, further investigations on the same experiments are underway—particularly the regulation of ET, salicylic acid (SA) and gibberellins (GAs) in biochar applied WBPH resistant and susceptible rice under WBPH attack.

## Conclusion

We found an optimum level of biochar (10%, w/w) that promotes plant physiological characteristics of both Cheongcheong and Nagdong rice varieties. Under the optimum level of biochar, Cheongcheong and Nagdong had contrasting responses to WBPH infestation. Interestingly, the Cheongcheong variety became susceptible to WBPH infestation, while the Nagdong gained resistance to WBPH infestation. Plant regulation of endogenous jasmonic acid levels in response to the priming effect of biochar could be one of the interrelated mechanisms explaining these results. The decrease in jasmonic acid levels under normal conditions and slight increase under WBPH infestation could indicate a regulated response to stress in Nagdong, which might explain the observed increase in resistance and plant growth parameters. In contrast, Cheongcheong significantly increased its jasmonic acid levels with the addition of the optimum biochar level under normal conditions. Cheongcheong jasmonic acid levels increased another two-fold in response to WBPH infestation. This may represent a maladaptive overproduction of jasmonic acid under stress, as these plants also exhibited decreases in plant growth parameters and WBPH resistance. However, the potential biological mechanisms which may have produced these results are not clear, and further investigations will be required in order to elucidate the relationship between jasmonic acid levels and WBPH infestation in various rice varieties. Understanding these results will be important for avoiding unfavorable impacts on crops, such as decreases in crop yields. The experimental results demonstrate that biochar addition may have differential effects depending on the variety of rice. Our results for the Nagdong and Cheongcheong varieties highlight this possibility, and these varieties would therefore be useful inclusions in future studies investigating the effects of biochar on plant responses to different types of stress. Due to the ambiguous plant responses to biochar amendment, intensive future research is necessary to explore the underlying biological mechanisms that dictate plant responses to biochar amendment.
